# Investigating Helium-Induced Thermal Conductivity Degradation in Fusion-Relevant Copper: A Molecular Dynamics Approach

**DOI:** 10.3390/ma18153702

**Published:** 2025-08-06

**Authors:** Xu Yu, Hanlong Wang, Hai Huang

**Affiliations:** 1 Key Laboratory of Material Physics, Ministry of Education, School of Physics, Zhengzhou University, Zhengzhou 450001, China; 2 International Joint Laboratory for Integrated Circuits Design and Application, Ministry of Education, School of Physics, Zhengzhou University, Zhengzhou 450001, China

**Keywords:** copper alloys, thermal transport, helium bubble, fusion reactor divertor, non-equilibrium molecular dynamics

## Abstract

Copper alloys are critical heat sink materials for fusion reactor divertors due to their high thermal conductivity (TC) and strength, yet their performance under extreme particle bombardment and heat fluxes in future tokamaks requires enhancement. While neutron-induced transmutation helium affects the properties of copper, the atomistic mechanisms linking helium bubble size to thermal transport remain unclear. This study employs non-equilibrium molecular dynamics (NEMD) simulations to isolate the effect of bubble diameter (10, 20, 30, 40 Å) on TC in copper, maintaining a constant He-to-vacancy ratio of 2.5. Results demonstrate that larger bubbles significantly impair TC. This reduction correlates with increased Kapitza thermal resistance and pronounced lattice distortion from outward helium diffusion, intensifying phonon scattering. Phonon density of states (PDOS) analysis reveals diminished low-frequency peaks and an elevated high-frequency peak for bubbles >30 Å, confirming phonon confinement and localized vibrational modes. The PDOS overlap factor decreases with bubble size, directly linking microstructural evolution to thermal resistance. These findings elucidate the size-dependent mechanisms of helium bubble impacts on thermal transport in copper divertor materials.

## 1. Introduction

Copper (Cu) and its alloys are prominent candidates for heat sink applications in fusion reactor divertors owing to their exceptional strength, superior thermal conductivity (TC), and high thermal stability [1,2,3]. However, these plasma-facing components within advanced magnetic confinement fusion systems must withstand extreme operational environments characterized by intense particle bombardment and substantial thermal fluxes [4,5,6]. In particular, future Tokamak designs, such as CFETR and DEMO, will impose even greater demands on divertor materials, requiring resilience against heat loads potentially reaching 20 MW·m^−2^ during extended steady-state operation [7,8]. Consequently, the thermophysical performance of Cu-based alloys under irradiation necessitates significant enhancement to meet these challenges.

High-dose neutron irradiation generates point defects, dislocation loops, and stacking-fault tetrahedra (SFTs) within materials, while simultaneously producing gaseous transmutation products (e.g., He atoms) through nuclear reaction [9,10,11]. These irradiation-induced alterations significantly degrade the thermal properties of copper and its alloys, ultimately shortening divertor service life [12,13]. For example, research into irradiation’s impact on thermal transport includes Zhang et al.’s investigation, which revealed that vacancies diminish the phonon TC of single-crystal copper via phonon scattering, with TC reduction proportional to vacancy concentration [14]. Ye et al. [15] demonstrated that tungsten doping (4 at.%) drastically reduces the TC of copper by approximately 80%, attributing this effect to significant differences in the electronic states of W and Cu atoms compared to vacancies or self-interstitial atoms, which impede electron transport. Furthermore, Fabritsiev et al. [16,17] observed a 25% decrease in TC for both CuCrZr and GlidCop Al-25 alloys at 573 K under neutron irradiation (2 dpa), identifying the nuclear transmutation effect as the primary contributor—responsible for 80% of this reduction. Nevertheless, the fundamental atomistic mechanisms through which irradiation damage, particularly neutron-induced transmutation helium, governs thermal transport degradation in Cu-based materials remain inadequately characterized.

Molecular dynamics (MD) simulations compute macroscopic system properties by statistically analyzing atomic positions and momenta within frameworks derived from statistical mechanics [18,19]. This approach can provide atomic-scale insights into the fundamental mechanisms by which irradiation damage degrades materials’ thermal transport characteristics [20,21]. Among the available computational techniques, the non-equilibrium MD (NEMD) method has become a principal tool for simulating thermal transport in nanomaterials and has yielded substantial advances in understanding thermal transport within material systems containing transmutation-produced He atoms [22,23]. For example, Petersson et al. [24] demonstrated that even low concentrations of interstitial helium in tungsten markedly reduce TC; however, vacancy–helium interactions that trap helium atoms can mitigate this degradation by reducing lattice distortion. Conversely, Sharma et al. [25] indicated that within nickel, increasing helium bubble diameter and internal helium concentration elevate both the temperature gradient and Kapitza resistance across the bubble region. Helium demonstrates negligible solubility in metallic systems, driving its precipitation into nanoscale bubbles within nuclear structural materials [26,27]. Although prior research has explored the influence of transmutation-produced helium on the structural, mechanical, and thermal properties of copper and its alloys [28,29], the atomistic mechanisms through which helium bubble dimensions govern thermal transport remain unresolved.

To address this knowledge gap, in this work, the NEMD method is utilized to examine the thermal transport behavior in copper materials from an atomistic perspective, with an emphasis on elucidating how helium bubbles impact thermal conductivity. We thoroughly investigate how helium bubble size affects the TC of copper materials. It is noteworthy that the He-to-vacancy ratio within bubbles of varying sizes remains constant, which allows us to isolate the effect of bubble size on thermal transport. This methodological approach aligns with established practices in the field, as evidenced by numerous prior studies [30,31]. The results highlight that larger helium bubbles more significantly diminish the TC of copper materials.

## 2. Simulation Methodology

All the MD simulations were conducted using the Large-scale Atomic/Molecular Massively Parallel Simulator code (LAMMPS, version 29 Aug 2024) [32], with atomic configurations visualized via the Open Visualization Tool software (OVITO, version 3.11.3) [33]. For modeling the Cu–He system, we adopted the well-established embedded atom method (EAM) potential developed by Kashinath et al. [34], which accounts for Cu–Cu, He–He, and Cu–He interatomic interactions. This potential reliably captures helium diffusion behavior and provides accurate predictions of both migration and formation energies for helium in face-centered cubic (fcc) copper [34].

**Figure 1:**
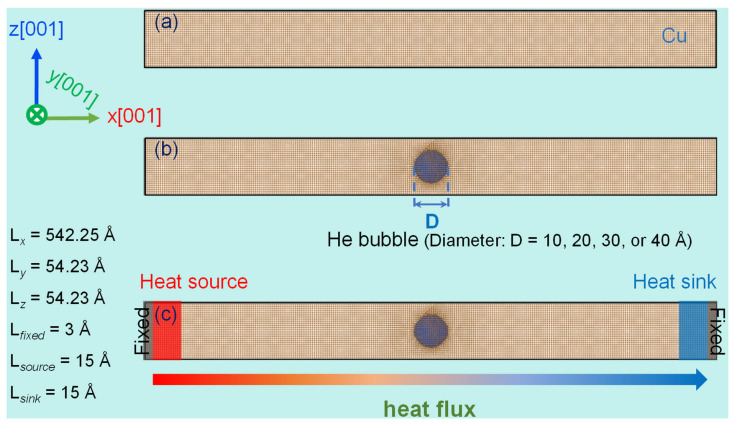
NEMD computational framework employed to analyze thermal transport in a copper system embedded with helium bubbles. (**a**) Atomic configuration of a pristine single-crystal copper structure. (**b**) Corresponding atomic model incorporating a helium bubble. (**c**) Schematic representation of the simulation domain, explicitly identifying the fixed boundary layers, heat source region, and heat sink region.

The simulation setup began with the creation of a single-crystal copper structure with dimensions of 150a_0_ × 15a_0_ × 15a_0_ (where a_0_ = 3.615 Å represents the Cu lattice constant [35,36]), containing approximately 135,000 atoms (see Figure 1a). The crystallographic orientations were aligned with the simulation axes, assigning [100], [010], and [001] directions to x, y, and z coordinates, respectively. A spherical void was then introduced at the system’s center, into which He atoms were randomly distributed to create an artificial bubble structure (see Figure 1b). Stress relaxation was performed via conjugate gradient energy minimization under zero external pressure. Periodic boundaries were maintained in all directions throughout simulations. Before thermal transport analysis, the system underwent 0.1 ns of NPT ensemble equilibration at 300 K and zero pressure using a Nosé–Hoover thermostat–barostat to achieve stability. Four distinct bubble diameters (10, 20, 30, and 40 Å) were investigated to assess size effects on thermal properties, maintaining a constant He-to-vacancy ratio of 2.5 [30]. For baseline comparison, a defect-free copper crystal was simulated under identical conditions.

To establish a thermal gradient, periodicity along the *x*-axis was disrupted by fixing outer layers (3.0 Å thickness) at both ends of the simulation cell. Adjacent to these fixed regions, paired 15.0 Å thick reservoirs generated a constant heat flux density of 5.5 × 10^9^ W·m^−2^ (see Figure 1c). Heat injection occurred at the left reservoir (source), propagated unidirectionally through the system, and dissipated at the right reservoir (sink). Atoms within the central adiabatic region evolved under NVE ensemble dynamics, while Langevin thermostats regulated reservoir temperatures. Following 0.2 ns of equilibration for system stabilization, temperature data were collected over 4 ns for time-averaging. Local temperatures were recorded within 12.7 Å thick bins along the *x*-axis to construct temperature profiles, enabling thermal transport efficiency evaluation in helium-bubble-containing copper. All simulations employed this consistent heat flux density methodology. The chosen flux magnitude (10^9^–10^10^ W·m^−2^) aligns with established NEMD methodologies for simulating extreme divertor thermal loads in fusion reactors [37,38]. Note that the elevated flux magnitude—exceeding physical conditions—intentionally enhances the temperature gradient resolution for analysis, consistent with comparable computational studies [37,38,39]. To mitigate stochastic uncertainties in defect configurations, each case was replicated three times, and the averaged results were reported.

## 3. Results and Discussion

**Figure 2:**
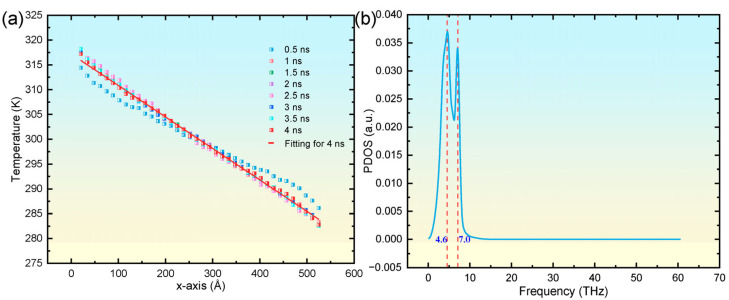
Temporal evolution of the spatial temperature distribution along the *x*-axis for a pristine single-crystal copper system at 300 K (**a**), alongside its corresponding PDOS spectrum at 4.0 ns (**b**).

To investigate the influence of helium bubbles, we first examined the thermal transport behavior of defect-free copper as a reference baseline. In the course of NEMD simulations, temperature field data were captured at consistent intervals of 0.5 ns to monitor the progression of thermal transport within a pristine copper crystal. Figure 2a illustrates that, at the 0.5 ns mark, there was a pronounced rise in temperature adjacent to the heat source, accompanied by a decline near the heat sink. Nonetheless, the temperature profile revealed substantial nonlinearity before 0.5 ns, suggesting that steady-state thermal transport had not been attained. After 1 ns, the temperature distribution achieved stability, manifesting an almost linear gradient along the *x*-axis. The temperature gradient was determined by fitting the temperature data at the 4 ns timepoint and was subsequently integrated with Fourier’s law [40]

(1) $$J=\kappa \frac{\mathrm{dT}}{\mathrm{dx}}$$

where J denotes the heat flux density, κ represents the TC, and dT/dx is the temperature gradient. Calculations yielded a TC of 7.98 W·m^−1^·K^−1^ for the defect-free copper crystal at 300 K, closely aligning with the 7.86 W·m^−1^·K^−1^ documented in a previous study [35] and thereby confirming the accuracy of the computational approach employed in this research. Typically, insights into the mechanisms of thermal transport can be gained indirectly by examining the phonon density of states (PDOS), as this provides a quantitative measure of variations in the phonon spectrum [41,42,43]. The PDOS is determined by applying the fast Fourier transform to the atomic velocity autocorrelation function (VACF) obtained from MD trajectories, as expressed in the equation [44,45]

(2) $$\mathrm{PDOS}(\omega )=\int_{-\infty }^{\infty }\;e^{-i\omega t}\mathrm{VACF}(t)\mathrm{dt}$$

where

(3) $$\mathrm{VACF}\left(t\right)=\frac{1}{N}\sum_{j=1}^{N}\;\left\langle v_{j}\left(0\right)v_{j}\left(t\right)\right\rangle$$

In this formulation, ω represents the phonon vibration frequency, N is the number of atoms within the analysis domain, v_j_(t) denotes the velocity vector of the jth atom at time t, and <∙∙∙> signifies the ensemble average. Consequently, the PDOS characteristics of the defect-free copper at 300 K were computed and are presented in Figure 2b. The analysis reveals that in defect-free coppers at 300 K, low-frequency phonon modes, predominantly distributed between 0 and 10 THz, dominate the PDOS spectrum. Spectral decomposition reveals a bimodal distribution featuring a more intense primary peak at 4.6 THz compared to the secondary peak at 7.0 THz. These findings are consistent with those reported in prior studies [39,46], thereby further validating the reliability of the simulation methodology employed in this work.

**Figure 3:**
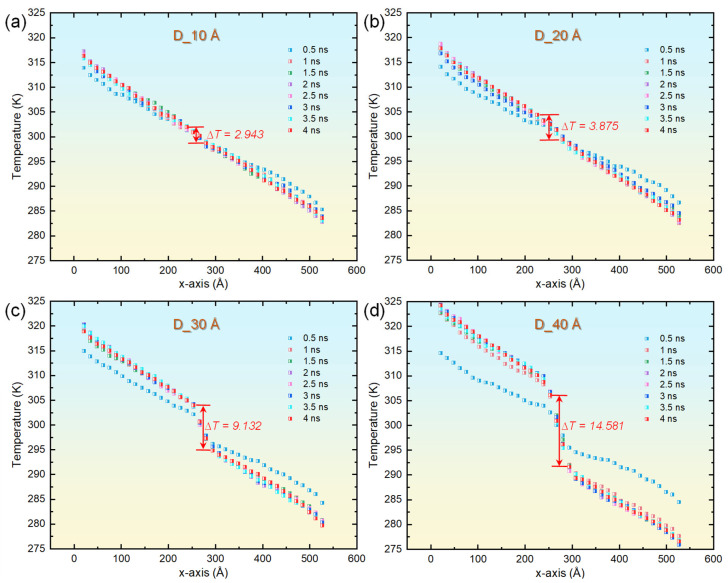
Temporal evolution of the spatial temperature distribution along the *x*-axis for single-crystal copper systems embedded with helium bubbles of varying diameters. (**a**) 10 Å. (**b**) 20 Å. (**c**) 30 Å. (**d**) 40 Å.

Numerous investigations have established that examining alterations in the PDOS serves as an effective method to elucidate the mechanisms by which microstructural characteristics, such as defects and interfaces, impact thermal transport properties [41]. This area of research has witnessed considerable advancements, as evidenced by various studies [47]. Leveraging this established framework, the current study undertakes a systematic examination of how the size of helium bubbles influences the TC within copper. Subsequently, we performed a detailed analysis to assess the impact of helium bubbles of different sizes on the thermal transport characteristics of copper. Figure 3 depicts the time-dependent changes in temperature distribution throughout the heat transport process in four distinct systems that incorporate helium bubbles. During the early phase of the simulation (at t = 0.5 ns) for each system containing helium bubbles, an elevation in temperature was noted in the vicinity of the heat source, concurrent with a reduction in temperature near the heat sink. This behavior mirrors that observed in defect-free copper. It is noteworthy that, at this juncture, the temperature profiles within the regions devoid of bubbles continued to display nonlinear traits, signifying that the system had not attained a state of steady heat conduction. As the simulation advanced beyond 1 ns, the temperature distribution achieved a stable configuration. Generally, an increase in the size of helium bubbles correlates with heightened fluctuations in the temperature distribution within the respective system. Within areas lacking bubbles, the temperature exhibited a distinct linear gradient along the *x*-axis. Conversely, regions housing helium bubbles experienced notable decreases in temperature. A quantitative assessment indicated temperature declines of 2.943 K, 3.875 K, 9.132 K, and 14.581 K for bubble diameters measuring 10 Å, 20 Å, 30 Å, and 40 Å, respectively. These findings unequivocally illustrate that the temperature gradient intensifies significantly as the size of the helium bubbles increases.

**Figure 4:**
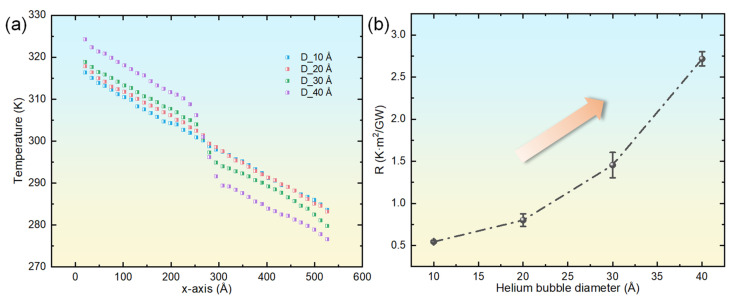
Instantaneous temperature distribution along the *x*-axis for single-crystal copper systems embedded with helium bubbles (diameters: 10–40 Å) at 4 ns (**a**), paired with the corresponding diameter-dependent Kapitza thermal resistance (**b**).

For comparative analysis, Figure 4a depicts the steady-state temperature distributions within copper systems that incorporate helium bubbles of diverse sizes. The examination reveals marked differences in the temperature gradient across the bubble regions, with the magnitude of this gradient escalating significantly as the bubble diameter increases. This effect may stem from the substantially lower thermal conductivity of the helium bubble regions (approximately 0.3 W·m^−1^·K^−1^ [48]) relative to the copper matrix, which induces pronounced thermal resistance effects. Additionally, as the helium bubble size expands, the temperatures adjacent to the system’s heat source and heat sink exhibit increasing and decreasing trends, respectively. Furthermore, this trend becomes more accentuated with larger helium bubble sizes. This behavior may be attributed to the heightened thermal resistance associated with larger helium bubbles, which impedes heat transfer along the *x*-axis, thereby complicating the dissipation of heat near the heat source and its absorption near the heat sink. When conceptualizing the helium-bubble-containing copper as a composite material with copper as the matrix and helium as the filler particles, Maxwell’s effective medium theory for spherical inclusions can be applied to determine the effective thermal conductivity [31]

(4) $$\frac{\kappa_{\mathrm{eff}}}{\kappa_{m}}=1+\frac{3\varphi }{\left(\frac{\frac{\kappa_{1}}{\kappa_{m}}+2}{\frac{\kappa_{1}}{\kappa_{m}}-\;1}\right)-\varphi }$$

where φ denotes the volume fraction of the void occupied by the helium bubble, and κ_m_ and κ_1_ represent the thermal conductivities of the perfect copper crystal and the helium bubble, respectively. For a vacuum void, κ_1_ = 0, whereas for a helium bubble, κ_1_ ≈ 0.3 W·m^−1^·K^−1^ [48]. Given that all simulations in this study maintained a constant He-to-vacancy ratio of 2.5, the computed effective thermal conductivities for copper systems with varying bubble diameters were found to be comparable. However, as illustrated in Figure 4a, significant temperature gradients are observed between systems with bubble diameters ranging from 10 Å to 40 Å, suggesting discrepancies in effective thermal conductivity. This inconsistency arises because the Maxwell model does not account for the phonon scattering effects induced by helium atoms in the proximity of the bubbles. To more accurately characterize the influence of helium bubbles on thermal transport properties, researchers have introduced the concept of Kapitza thermal resistance (viz., the reciprocal of interfacial thermal conductance) to quantify the abrupt changes in temperature gradient near the bubble regions [25]. The Kapitza resistance is defined by

(5) $$R=\frac{\Delta T}{J}$$

where ΔT signifies the temperature difference across the helium bubble region (as depicted in Figure 4a), and J represents the heat flux density along the direction of thermal transport. Figure 4b displays the Kapitza thermal resistance for copper systems containing helium bubbles of different diameters. The findings indicate a progressive increase in Kapitza resistance as the bubble diameter enlarges. Notably, the increment is relatively modest for bubbles with diameters between 10 Å and 20 Å but becomes more substantial for diameters exceeding 20 Å.

**Figure 5:**
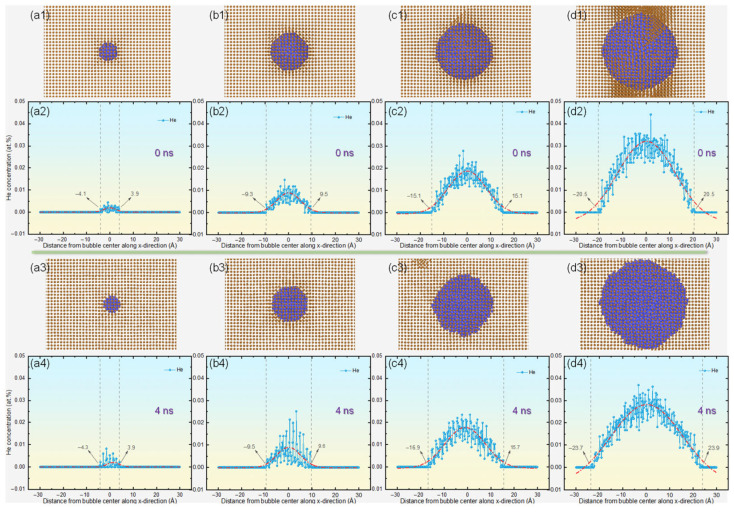
Evolution of helium bubble configurations and their associated concentration profiles along the *x*-axis during thermal transport simulations in copper, comparing initial (0 ns) and final (4 ns) states for varying bubble diameters: (**a**) 10 Å, (**b**) 20 Å, (**c**) 30 Å, and (**d**) 40 Å. Panels (**a1**–**d1**) and (**a3**–**d3**) depict the spatial distribution of helium bubbles at both timepoints, while (**a2**–**d2**) and (**a4**–**d4**) quantify the corresponding helium concentration gradients across the simulated copper matrices.

Fluctuations in Kapitza resistance are likely linked to the evolution of atomic configurations at the microscopic scale within the system. To investigate the underlying causes, we meticulously analyzed the spatial arrangement of He atoms along the *x*-axis in systems featuring helium bubbles with diameters ranging from 10 to 40 Å. By designating the x-coordinate of each bubble’s center as the reference point, we assessed the distribution of He atoms by determining their relative abundance at various distances. Figure 5 illustrates the concentration of helium along the *x*-axis at both the initial (0 ns) and final (4 ns) stages of the thermal transport simulations in copper, incorporating bubbles of different sizes. A comparison of the helium distribution between the initial and final stages of the simulations reveals that larger bubbles facilitate greater outward diffusion of helium atoms, signifying their infiltration into the copper lattice [31,49]. In systems with smaller bubbles (e.g., 10 Å and 20 Å), there is negligible outward diffusion, indicating minimal distortion of the surrounding copper lattice. This observation aligns with the modest increase in Kapitza resistance depicted in Figure 4b. Conversely, for bubbles that are 30 Å in diameter, helium atoms begin to extend beyond the initial bubble boundaries, suggesting increased local lattice distortion. This phenomenon is more pronounced with a 40 Å bubble, where helium diffusion reaches approximately 23.7 Å from the center, displacing adjacent Cu atoms and inducing significant lattice deformation. Such atomic-level disruptions markedly intensify phonon scattering, thereby hindering phonon transmission and leading to a rapid escalation in Kapitza resistance. These results demonstrate a direct relationship between the size of helium bubbles and the Kapitza resistance, offering essential insights into the mechanisms governing thermal transport in copper systems containing helium bubbles.

**Figure 6:**
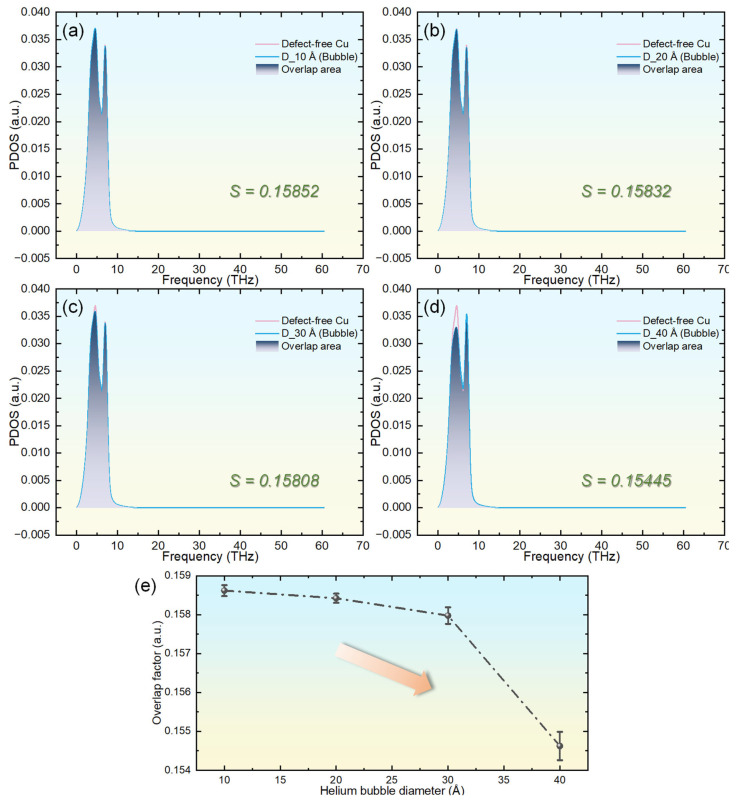
Comparison of PDOS spectra between pristine copper and helium-embedded copper systems with bubble diameters of (**a**) 10 Å, (**b**) 20 Å, (**c**) 30 Å, and (**d**) 40 Å. Panel (**e**) quantifies the diameter-dependent PDOS overlap factor between the two systems.

To further substantiate our analysis of helium’s effect on phonon thermal transport within the crystal lattice, we computed the PDOS for each system, as illustrated in Figure 6a–d. Additionally, to quantitatively evaluate the similarity between phonon modes in systems with helium bubbles and those in pristine copper, we introduced a PDOS overlap factor [39], defined as

(6) $$S=\int_{0}^{\infty }\;\min\left\{P_{0}(\omega ),P_{\mu }\left(\omega \right)\right\}d\omega$$

where P_0_(ω) denotes the PDOS of defect-free copper and P_μ_(ω) represents that of helium-bubble-containing systems. According to Figure 6a−d, helium bubbles with diameters of 10 Å and 20 Å cause only slight modifications to the PDOS of copper. This finding clarifies why these systems exhibit thermal conductivity akin to that of defect-free copper and maintain low Kapitza resistance. In contrast, bubbles larger than 30 Å lead to a significant drop in the low-frequency PDOS peak, coupled with a rise in the high-frequency peak. For instance, in the system with a 40 Å bubble, the low-frequency peak intensity decreases from 0.03697 to 0.03300, while the high-frequency peak intensity increases from 0.03389 to 0.03543. This shift occurs because low-frequency phonons, which are tied to long-wavelength acoustic modes, dominate thermal transport [50]. The reduction suggests that helium diffusing outward from the bubbles disturbs the copper lattice’s periodicity, confining these phonons near the bubble regions and thus hindering heat conduction, which explains the elevated Kapitza resistance. Conversely, the increased high-frequency peak arises from the mass contrast between He and Cu atoms, as lighter helium impurities introduce localized high-frequency vibrational modes, boosting their density [51]. Figure 6e shows the PDOS overlap factor’s variation between helium-bubble-containing systems and defect-free copper, with a declining overlap factor as bubble size grows, mirroring the trend in Kapitza resistance and affirming that phonon-based analysis effectively reveals the influence of helium bubbles on the thermal transport properties of copper.

## 4. Conclusions

In summary, this study investigated how helium bubble size (10–40 Å diameter) influences the thermal conductivity of copper using NEMD simulations. Simulations first established a defect-free copper baseline (TC = 7.98 W·m^−1^·K^−1^ at 300 K), with PDOS analysis showing dominant low-frequency modes (0–10 THz), validating the methodology. The results demonstrate that larger bubbles progressively reduce TC, with temperature drops near bubbles intensifying from 2.943 K (10 Å) to 14.581 K (40 Å). Steady-state analyses reveal significantly amplified temperature gradients across bubble regions as diameters increase, attributed to helium’s low intrinsic conductivity (~0.3 W·m^−1^·K^−1^), which induces interfacial thermal resistance. Kapitza resistance exhibits nonlinear growth, rising modestly below 20 Å but substantially above 30 Å. This trend correlates with helium diffusion patterns: bubbles >30 Å show pronounced outward helium migration (reaching 23.7 Å from the center for 40 Å bubbles), displacing Cu atoms and distorting the lattice, thereby intensifying phonon scattering. PDOS analysis confirms bubbles >30 Å suppress low-frequency phonons (critical for heat conduction) by up to approximately 11%, while enhancing high-frequency modes due to mass contrast effects. Correspondingly, the PDOS overlap factor with pristine copper decreases by 0.13–2.57% for larger bubbles, directly mirroring the trends for Kapitza resistance. These findings establish that size-dependent helium diffusion drives lattice disruption, phonon confinement, and interfacial resistance, providing atomistic insights into thermal degradation mechanisms in copper alloys for fusion applications.

## Data Availability

The data that support the findings of this study are available from the corresponding author upon reasonable request.
